# A Giant Solitary Fibrous Tumor of the Pleura Exceeding 15 cm Classified as Low Risk: A Rare Case Report

**DOI:** 10.70352/scrj.cr.26-0085

**Published:** 2026-04-28

**Authors:** Yuki Ichikawa, Keisuke Yokota, Kensuke Iguchi, Shin Hosokawa, Emi Hagui, Kensuke Chiba, Ryuji Nakamura, Tsutomu Tatematsu, Katsuhiro Okuda

**Affiliations:** Department of Thoracic and Pediatric Surgery, Graduate School of Medical Sciences and Medical School, Nagoya City University, Nagoya, Aichi, Japan

**Keywords:** solitary fibrous tumors of the pleura, giant tumor, thoracic surgery

## Abstract

**INTRODUCTION:**

Solitary fibrous tumors of the pleura (SFTPs) are rare mesenchymal neoplasms that are usually benign but may occasionally recur or undergo malignant transformation. Tumor size is an important prognostic factor; lesions larger than 10 cm are associated with recurrence and poor outcomes, and tumor size ≥15 cm is considered as a risk factor in prognostic models. Histologically, benign SFTPs of ≥15 cm in size are extremely uncommon.

**CASE PRESENTATION:**

A 48-year-old male presented with exertional dyspnea and chest tightness. Chest CT revealed a giant mediastinal mass measuring 138 × 91 × 129 mm. The encapsulated tumor originated from the pleura and was completely resected via a thoracotomy. Histological examination revealed spindle cells with hyalinization and calcification, without necrosis or mitotic figures. Immunohistochemical staining was positive for CD34 and STAT6, confirming a diagnosis of SFTP. The resected specimen measured 185 × 80 × 145 mm, with a low Ki-67 labeling index of 3%, and was classified as low risk according to the 4-variable Demicco risk stratification model.

**CONCLUSIONS:**

We report a rare case of a histologically benign but giant SFTP exceeding 15 cm in diameter. Although classified as low risk, large tumor size itself may reflect biological aggressiveness and be associated with recurrence or malignant transformation. Therefore, careful long-term follow-up is required.

## Abbreviations

HPF
: high-power fields
SFTPs
: solitary fibrous tumors of the pleura

## INTRODUCTION

Solitary fibrous tumors are rare mesenchymal neoplasms, with an estimated incidence of 2.8 per 100000 individuals, although pleural lesions represent only a subset of these tumors.^1)^ Since 1931, only 900 cases have been reported worldwide in the relevant literature.^2,3)^ Most SFTPs are histologically benign and tend to have a favorable clinical course following surgical resection. However, approximately 12% of SFTPs are malignant, and benign tumors may sometimes follow an aggressive clinical course.^4,5)^ In rare cases, benign SFTPs have been reported to recur multiple times.^6,7)^ Most SFTPs are <10 cm in size, but they can occasionally grow significantly larger and compress adjacent organs, leading to clinical symptoms. Harrison-Phipps et al. reported that the median tumor size was 4.5 cm for benign SFTPs and 12.0 cm for malignant ones.^8)^ Lahon et al. found that benign SFTPs had an average size of 6.4 cm, while malignant cases averaged 13.4 cm, a statistically significant difference (p < 0.0001).^3)^ In addition, Tapias et al. and Gold et al. identified a tumor size >10 cm as an independent risk factor for recurrence and a poor prognosis.^9,10)^ In the Demicco risk model, tumor size ≥15 cm contributes to a higher risk score.^11)^ These findings suggest that histologically benign SFTPs ≥15 cm are rare and warrant special attention owing to their potential impact on the prognosis. We herein report a rare case of a giant SFTP >15 cm in size, which was histologically benign, and describe its clinicopathological features along with a review of the relevant literature.

## CASE PRESENTATION

A 48-year-old male presented to our hospital with complaints of exertional dyspnea and a sensation of chest tightness. He had been experiencing these symptoms for approximately 1 month and had consulted multiple physicians before being referred to our hospital. The patient’s medical history was unremarkable. He had a significant smoking history (40 cigarettes per day for 7 years), and had stopped smoking 17 years prior to his presentation.

**Fig. 1:**
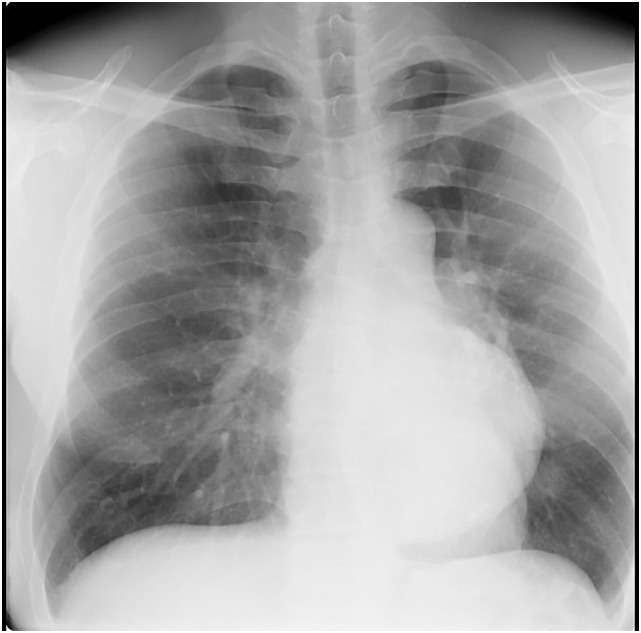
Chest X-ray findings of the patient. Chest X-ray film showed a mass in the left hilar region extending toward the lower mediastinum.

Chest radiography performed at a previous facility revealed a mass in the left hilar region (**Fig. 1**). Transthoracic echocardiography demonstrated a mass adjacent to the heart. Therefore, he was referred to our department of thoracic surgery for further evaluation and surgical resection.

**Fig. 2:**
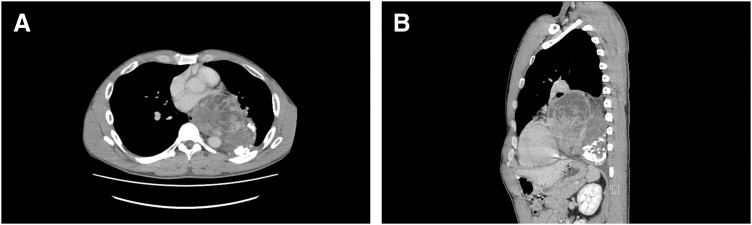
Chest CT findings of the patient. Chest CT scan showed a well-defined giant mass is observed extending from the middle to posterior mediastinum on the left side. The lesion showed heterogeneous enhancement with areas of hyalinization and calcification. (**A**) axial view. (**B**) Sagittal view.

Chest CT revealed a large mediastinal mass measuring 138 × 91 × 129 mm, extending from the middle to the posterior mediastinum (**Fig. 2**). Contrast-enhanced CT showed a well-circumscribed mass with heterogeneous enhancement and partial calcification. The heart was displaced by the tumor; however, no clear invasion of adjacent structures, including major vessels, was observed. The differential diagnosis based on imaging findings included a neurogenic tumor, hemangioma, and teratoma. Although the tumor origin could not be clearly determined preoperatively, a solitary fibrous tumor was also considered. MRI was additionally performed; however, it did not provide additional diagnostic value, and the differential diagnosis remained unchanged. Although malignancy could not be completely excluded, the patient was considered for surgical resection based on the imaging findings and the patient’s symptoms. Preoperative biopsy was not performed due to the risk of tumor seeding and bleeding. The operative strategy was to assess resectability intraoperatively and proceed with complete resection if feasible.

Surgery was performed via thoracotomy through an initial 10-cm incision. The tumor originated from the parietal pleura and was adherent to the descending aorta, but no invasion of adjacent organs, including the esophagus, diaphragm, or chest wall, was observed. After confirming that complete resection was feasible, the incision was extended and the tumor was removed. During dissection around the left inferior pulmonary vein, the pericardium was opened; however, an appropriate dissection plane was re-identified, allowing safe continuation of the procedure. The operative time was 350 minutes and the estimated blood loss was 390 g. Pathological examination confirmed negative surgical margins (R0 resection).

**Fig. 3:**
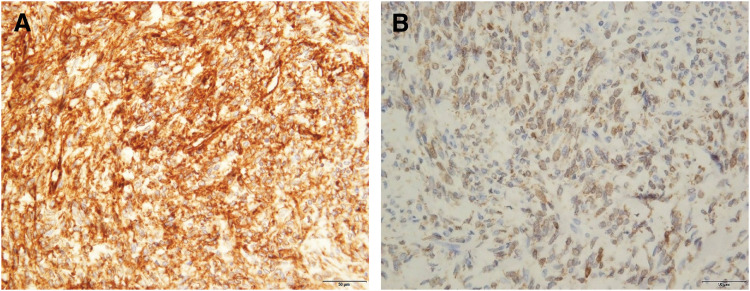
Immunohistochemical findings of the tumor. (**A**) Diffuse cytoplasmic positivity for CD34 in the spindle cells. (**B**) Nuclear positivity for STAT6, confirming the diagnosis of solitary fibrous tumor (Original magnification ×200).

**Fig. 4:**
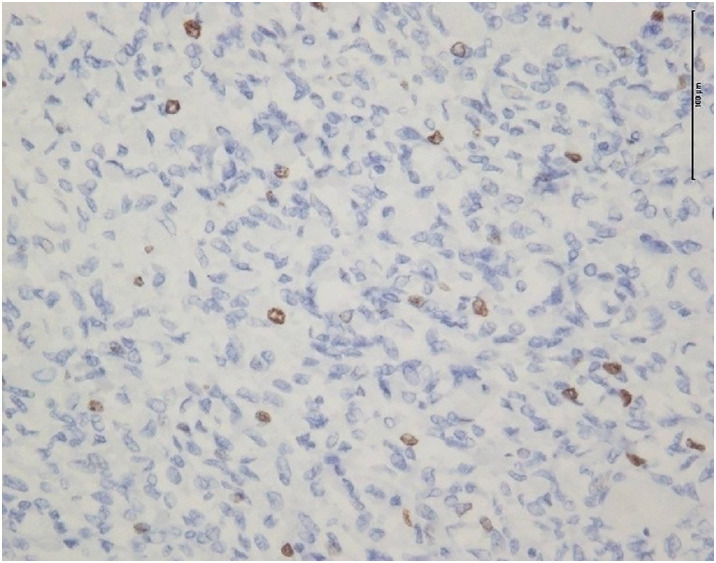
Immunohistochemical staining for Ki-67. The Ki-67 labeling index was approximately 3% (Original magnification ×200).

A histopathological examination revealed densely proliferating spindle-shaped cells accompanied by numerous blood vessels, hyalinization, and calcification. No malignant features such as nuclear atypia, mitotic activity, necrosis, or hemorrhage were observed. Immunohistochemically, the tumor cells were positive for CD34 and STAT6, which was consistent with the diagnosis of SFTP (**Fig. 3**). The resected tumor measured 185 × 80 × 145 mm, and the Ki-67 labeling index was low at 3% (**Fig. 4**).

According to the 4-variable Demicco risk stratification model, the tumor was classified as low risk based on the patient’s age, tumor size, mitotic count, and the absence of necrosis (**Table 1**).

**Table 1:** Risk assessment according to the 4-variable Demicco model in this case

Variable	Finding	Score
Age	48 years	0
Tumor size	18.5 cm	3
Mitotic count	0/10 HPFs	0
Tumor necrosis	Absent	0
Total score		3
Risk category		Low risk

The postoperative course was uneventful, and no recurrence was observed 1 year after surgery. The patient is currently under close follow-up. Long-term follow-up with chest CT every 6 months for at least 10 years is planned, considering the risk of late recurrence.

## DISCUSSION

Most SFTPs are histologically benign and tend to follow an indolent course after complete surgical resection.^1,5,6)^ The histological classification of benign or malignant SFTP is commonly based on the criteria proposed by England et al., which include the following 4 features: 1) increased cellularity, 2) more than 4 mitoses per 10 HPF, 3) nuclear pleomorphism, and 4) hemorrhage or necrosis. Tumors exhibiting any of these features are classified as malignant.^6)^ However, even benign tumors may recur or metastasize.^12,13)^ The reported 5-year survival rates are approximately 89% for benign SFTPs and 56% for malignant SFTPs.^8,10)^ Disease-free survival has also been shown to correlate with tumor size and resection completeness. Malignant SFTP tends to present with larger tumors, often >10 cm in size.^8,9)^

In the present case, although histologically benign, the tumor measured over 15 cm and caused compression-related symptoms due to its massive size, which is an extremely rare finding. Reports of SFTPs measuring ≥15 cm in size are scarce, making this case clinically valuable. For instance, Crnjac et al. reported a benign SFTP measuring >22 cm, highlighting that even benign tumors can cause significant mass effect symptoms.^14)^ Furthermore, Briselli et al.’s review of 360 cases revealed that benign SFTPs larger than 15 cm were exceedingly uncommon,^4)^ underscoring the rarity of giant benign tumors such as the present case.

Tumor size has been shown to be correlated with recurrence and the prognosis. The risk stratification model proposed by Demicco et al., adopted in the 2020 WHO classification, evaluates risk based on patient age, tumor size, mitotic activity, and tumor necrosis.^11)^ Although a tumor size ≥15 cm contributes to the overall risk score, the final risk category is determined by the weighted combination of multiple variables rather than tumor size alone. In this case, based on the patient’s age, tumor size (≥15 cm), lack of mitotic activity (0/10 HPF), and absence of necrosis, the tumor was classified as low risk in the 4-variable model.

Recent studies have also implicated immunohistochemical markers such as CD34 loss, the overexpression of p53, and elevated Ki-67 as indicators of potential malignancy in SFTPs. In particular, the increased expression of Ki-67 has been reported in recurrent cases.^10,15)^ In the present case, the tumor was positive for CD34 and STAT6, with a Ki-67 labeling index of 3%, suggesting a favorable prognosis. Nevertheless, there have been reports of tumors initially deemed benign that later recurred or underwent malignant transformation during long-term follow-up, necessitating continued vigilance.

Given that a tumor size exceeding 15 cm is a risk factor for recurrence and malignant transformation, close and prolonged follow-up is essential. This case represents a rare example of a giant benign SFTP, highlighting the need for further case accumulation and molecular research to refine risk stratification in the future.

## CONCLUSIONS

We report a rare case of a giant SFTP ≥15 cm in size, which was histologically benign and completely resected. Although classified as low risk according to the Demicco model, a large tumor size itself is a known risk factor for recurrence and malignant transformation. Therefore, even histologically benign giant SFTPs require careful long-term follow-up.

## DECLARATIONS

### Availability of data and materials

Data sharing is not applicable to this article, because datasets were neither generated nor analyzed for case reports.
